# Micro-Discipline: A Process Model of Behavioural Regulation and Character Formation

**DOI:** 10.3390/bs16060879

**Published:** 2026-06-01

**Authors:** Åke Elden

**Affiliations:** Department of Research, NLA University College, 0176 Oslo, Norway; akeeld@nla.no

**Keywords:** micro-discipline, character formation, agency, self-regulation

## Abstract

Research on personality and behaviour has established that individuals exhibit relatively stable patterns of conduct across time, commonly described in terms of trait dimensions such as conscientiousness. At the same time, self-regulation and habit research have identified mechanisms involved in behavioural initiation, persistence, and automatization. Despite these advances, existing frameworks do not adequately specify the intermediate processes through which behavioural continuity is maintained across everyday contexts. This article introduces the concept of micro-discipline to address this gap. Micro-discipline refers to recurrent low-level acts of behavioural regulation that preserve continuity between intention and action under ordinary conditions of friction, including returning attention to a task, sustaining effort despite resistance, modulating minor impulses, and completing small obligations that might otherwise be deferred. The central claim is that these repeated regulatory acts constitute a distinct and temporally cumulative process through which behavioural patterns are stabilised and, over time, modified. Drawing on personality theory, self-regulation research, and related process-based approaches, the article develops a conceptual model explaining how such micro-regulatory processes bias the recurrence, persistence, and interruption of behavioural states, thereby contributing to trait stabilisation and trait change. By clarifying this intermediate process layer, the framework provides a more precise account of how local regulatory acts scale into durable patterns of behaviour. It further offers implications for understanding personality development, the maintenance of goal-directed behaviour, and the conditions under which intentional behavioural change succeeds or fails.

## 1. Introduction

Research on personality and behaviour has consistently demonstrated that human conduct exhibits recognisable forms of stability across time. Trait theories describe relatively enduring dispositions that organise patterns of thought, action, and response across situations, while contemporary personality research has shown that traits such as conscientiousness reliably predict occupational performance, health behaviour, persistence, and interpersonal functioning (McCrae & Costa, 2008; DeYoung, 2015). At the same time, research on self-regulation has clarified many of the processes involved in initiating, sustaining, and adjusting behaviour under changing situational demands.

Despite these advances, an important explanatory problem remains insufficiently clarified. Existing frameworks effectively describe stable behavioural tendencies and identify mechanisms of behavioural control, yet they often leave unresolved how coherence in everyday conduct is practically sustained across time. Most human behaviour depends less on exceptional acts of willpower than on recurrent acts of low-level regulation: redirecting attention after distraction, sustaining effort despite resistance, modulating competing impulses, and completing tasks that could easily be deferred. These processes are central to the maintenance of organised conduct, yet their cumulative role in shaping behavioural stability remains conceptually diffuse across the existing literature.

This article introduces the concept of micro-discipline to clarify this intermediate level of behavioural organisation. Micro-discipline refers to recurrent low-level regulatory enactments through which individuals preserve continuity between intention and action under ordinary conditions of friction. The argument is not that these regulatory mechanisms are entirely novel, but that their integrative and cumulative role has not been sufficiently theorised as a distinct explanatory process. Repeated acts of attentional return, effort continuation, impulse modulation, and behavioural completion gradually influence the recurrence, persistence, and consolidation of particular behavioural states. Over time, these regulatory patterns contribute to the emergence, reinforcement, or modification of more enduring forms of conduct. Behavioural stability, on this account, is not merely the expression of underlying traits but an ongoing achievement sustained through repeated regulatory maintenance.

The framework developed here integrates insights from personality psychology, self-regulation research, process-based approaches to behaviour, and virtue theory. Trait models successfully describe relatively stable behavioural regularities but often function primarily at the level of descriptive outcomes. Research on habits and self-control explains important mechanisms of automatization, impulse management, and goal pursuit, yet frequently concentrates either on cue-driven repetition or episodic conflict regulation. Virtue ethics, meanwhile, provides sophisticated normative accounts of character formation while often leaving the underlying behavioural processes comparatively underdeveloped. Across these traditions, an explanatory gap persists between isolated actions and stable dispositions.

Recent developments in personality science suggest that this missing explanatory layer is theoretically significant. Process-oriented approaches increasingly conceptualise traits as distributions of enacted states unfolding across situations and time (Fleeson, 2001; Jayawickreme et al., 2021). Longitudinal research similarly indicates that personality development is typically gradual and associated with accumulated behavioural adjustments rather than abrupt transformation (Roberts et al., 2006; Hudson & Fraley, 2015). These perspectives imply that enduring behavioural organisation emerges through repeated regulatory activity operating at relatively fine temporal scales.

The present account develops this implication more explicitly. The central claim is that character formation depends partly on cumulative processes of behavioural maintenance situated between momentary action and stable disposition. Behavioural coherence cannot be adequately explained either through traits alone or through isolated decisions. Rather, it emerges through iterative patterns of regulation distributed across ordinary life.

### Methodological Approach: Conceptual Synthesis

This article adopts a conceptual synthesis approach integrating insights from personality psychology, self-regulation research, and virtue theory. Rather than providing a systematic review, the analysis reconstructs an insufficiently specified explanatory layer linking momentary action and stable dispositions. The literature is selected according to its relevance for understanding intermediate processes of behavioural regulation, and the argument proceeds through conceptual clarification, comparative analysis, and theoretical integration. The contribution is primarily explanatory, aiming to refine the conceptual architecture through which behavioural regulation and character formation are understood.

The article proceeds as follows. Section 2 examines the problem of behavioural scale and the relation between actions, regulatory processes, and stable dispositions. Section 3 defines micro-discipline and distinguishes it from adjacent constructs including habit, self-control, and conscientiousness. Section 4 develops an account of the cumulative mechanisms through which micro-regulatory enactments shape behavioural trajectories across time. Section 5 clarifies the relation between behavioural organisation and moral evaluation. Section 6 advances an ecological account of micro-discipline as a person–environment regulatory system. Section 7 considers the implications of this framework for understanding the temporal structure of agency.

The framework is intended to function as a bridge between descriptive accounts of personality and normative accounts of character by specifying the behavioural processes through which stability is enacted and maintained. Related concerns have also emerged in debates surrounding the stability of character and the significance of situational variability in moral psychology (Doris, 2002).

## 2. Character Between Action and Structure: The Problem of Behavioural Scale

Accounts of character have traditionally operated at two primary levels of analysis. The first concerns individual actions, where ethical theory evaluates conduct in terms of right and wrong, virtue and vice. The second concerns relatively stable dispositions. Within virtue ethics, character is understood as an enduring configuration of evaluative tendencies that reliably shapes behaviour across circumstances (Annas, 2011; Snow, 2010).

Between these levels lies a comparatively underdeveloped domain: the fine-grained regulatory processes through which momentary actions gradually consolidate into enduring patterns of conduct. This gap reflects what may be described as a problem of behavioural scale. Ethical theory frequently analyses agency at the level of deliberative choice, whereas personality psychology typically focuses on relatively stable traits. Yet much of everyday behaviour unfolds within an intermediate temporal layer composed of recurrent forms of regulation that organise conduct across time. Understanding character formation therefore requires closer attention to how behavioural regulation operates between isolated action and durable disposition. 

### 2.1. Actions, Traits, and the Architecture of Behaviour

In Aristotelian virtue ethics, character emerges through habituation. Repeated actions gradually shape dispositions that influence perception, judgment, and conduct (Aristotle, 1999, II.1, 1103a14–25). Contemporary virtue theorists retain this emphasis. Annas (2011) argues that virtues develop through practice in which agents acquire stable forms of evaluative judgment, while Snow (2010) emphasises the role of enduring motivational and perceptual orientations in guiding behaviour across contexts. 

At the same time, philosophical accounts of habituation rarely specify the behavioural processes through which repeated actions accumulate into stable dispositions. Habituation is commonly invoked as a developmental mechanism, yet the underlying regulatory dynamics often remain conceptually abstract.

A similar pattern appears within personality psychology. Trait models describe behavioural stability in terms of dispositional structures such as conscientiousness and neuroticism (McCrae & Costa, 2008; DeYoung, 2015). These frameworks successfully capture recurring patterns of behaviour and experience across situations, but they frequently function more as descriptive summaries than as detailed accounts of how behavioural regularities are generated, stabilised, and maintained across time. 

Across both traditions, explanatory emphasis tends to fall on relatively stable outcomes. Philosophy describes the normative organisation of character, while psychology describes the descriptive structure of traits. Less clearly articulated are the regulatory processes through which behavioural stability is progressively enacted and sustained.

### 2.2. Situation–Behaviour Dynamics and Behavioural Signatures

Developments in personality science suggest that this intermediate regulatory layer is essential for understanding character formation. The social-cognitive framework proposed by Mischel and Shoda (1995) argues that personality coherence emerges not only from global traits but from stable situation–behaviour contingencies, often described as behavioural signatures. 

From this perspective, individuals display characteristic if–then patterns of behaviour across contexts. Rather than behaving uniformly, they respond systematically to particular classes of situations. An individual may remain composed under routine pressure yet react strongly to perceived social rejection. Behavioural consistency therefore arises through recurring configurations of situational cues and patterned responses.

This perspective highlights an important point: behavioural stability is not solely a function of abstract dispositional structure but depends on recurrent forms of context-sensitive regulation. Character coherence, in this sense, reflects the consolidation of patterned responses across repeated situations.

### 2.3. Personality Development and Incremental Behavioural Change

Research on personality development further supports this process-oriented interpretation. Longitudinal studies show that personality traits typically change gradually across the life course and that such changes often reflect accumulated behavioural adjustments rather than abrupt psychological transformation (Roberts et al., 2006). 

For example, increases in conscientiousness are frequently associated with changes in roles, responsibilities, and behavioural routines. Small shifts in everyday organisation, such as maintaining schedules, reliably completing obligations, or regulating impulses, can accumulate into more stable forms of conduct over time. Hudson and Fraley (2015) similarly demonstrate that intentional behavioural adjustments can produce measurable personality change. 

These findings suggest that both trait stability and trait development emerge through repeated regulatory activity operating at relatively fine temporal scales. This interpretation is consistent with DeYoung’s Cybernetic Big Five Theory, which conceptualises personality traits as parameters of goal-directed regulatory systems (DeYoung, 2015). Within this framework, behavioural coherence emerges through ongoing processes of monitoring, feedback, and adjustment. 

Taken together, these developments indicate that stable behavioural organisation depends on recurrent regulatory processes operating below the level of global trait descriptions.

### 2.4. Habit Formation and Repetition

Research on habit formation provides additional insight into how repeated behaviours generate stable patterns. Habits are commonly understood as behaviours that become automatically triggered by contextual cues through repetition (Wood & Rünger, 2016). Such processes can produce durable regularities that persist even in the absence of deliberate intention. 

However, habit formation does not fully account for many forms of behaviour central to character development. Activities such as sustaining a writing practice, fulfilling obligations, regulating emotional responses, or maintaining effort during difficult tasks often depend on continued regulation rather than complete automaticity. These forms of conduct require repeated acts of attention, adjustment, and effort rather than purely cue-driven execution.

In such domains, behavioural stability emerges not primarily through automatization but through ongoing low-level regulation distributed across time.

### 2.5. The Intermediate Layer of Behavioural Regulation

The convergence of these perspectives points toward an intermediate layer of behavioural organisation situated between isolated actions and stable traits. This layer consists of recurrent regulatory processes through which individuals preserve continuity between intention and action under ordinary conditions. 

These processes involve adjustments in attention, effort, impulse modulation, and task completion. Individually they may appear minor, yet through repetition they accumulate into behavioural trajectories that gradually stabilise into more enduring forms of conduct.

This intermediate domain may be understood as the microstructure of behavioural regulation. Micro-discipline refers to the stabilisation of this microstructure through repeated enactment. Behavioural patterns, in this view, are not merely expressed but progressively constructed and reinforced through ongoing regulation. Behavioural continuity is also shaped through the interaction between goal-directed processes and habitual responses (Wood & Neal, 2007).

Behaviour therefore cannot be adequately explained solely through discrete decisions or fixed dispositions. It is sustained through temporally extended regulatory processes operating at a finer behavioural scale. Recognising this level of organisation clarifies how momentary acts contribute to the emergence and persistence of more durable behavioural patterns.

### 2.6. What Micro-Discipline Explains That Adjacent Constructs Do Not

A natural objection is that the behavioural domain described here is already addressed by theories of habit, self-control, executive function, trait regulation, or virtue habituation. Each of these frameworks captures an important dimension of behavioural organisation. However, none isolate the specific explanatory role developed in this article. 

Habit theory explains how repeated behaviour becomes increasingly automatic through contextual cueing. Self-control research explains processes of impulse inhibition and conflict management. Trait theory describes relatively stable behavioural tendencies and, in some cases, their regulatory parameters. Virtue ethics explains how character becomes normatively organised through habituation and practical judgment.

What these frameworks do not isolate with sufficient precision is the cumulative role of recurrent low-level regulation in sustaining continuity between intention and action under ordinary conditions of friction.

The concept of micro-discipline is introduced to specify this intermediate explanatory layer. Its contribution lies not in identifying entirely novel psychological events, but in integrating dispersed mechanisms into a clearer account of how behavioural stability is maintained and how more enduring forms of conduct gradually emerge. Micro-discipline is therefore best understood not as a competing theory, but as a clarifying framework linking momentary regulatory processes to longer-term behavioural organisation.

## 3. Conceptual Delimitation and the Structure of Micro-Discipline

Two clarifications are necessary at the outset. First, micro-discipline should not be understood as conscientiousness under another name. Conscientiousness is a dispositional construct describing relatively stable tendencies toward orderliness, persistence, and dependability. Micro-discipline instead refers to the regulatory processes through which such behavioural regularities are sustained, reinforced, or weakened across time. The distinction is therefore explanatory rather than terminological.

Second, the concept should not be interpreted in moralised terms. Micro-discipline is not an endorsement of productivity norms, nor a framework for attributing personal failure under conditions where behavioural coherence is undermined by instability, scarcity, excessive cognitive load, or structural constraint. The framework is primarily descriptive. Its purpose is to identify and clarify a specific layer of behavioural organisation rather than to assign moral value to behavioural consistency itself.

Micro-discipline refers to recurrent low-level regulatory processes through which individuals preserve continuity between intention and action under ordinary conditions of friction. These processes involve adjustments in attention, effort, impulse modulation, sequencing, and completion rather than major decisions or exceptional moments of conflict. Their function is to sustain organised conduct in the face of distraction, fatigue, competing demands, and minor resistance. Micro-discipline is therefore best understood not as a trait or isolated act of self-control, but as an ongoing process of behavioural stabilisation distributed across time.

The concept is intentionally narrower than a general theory of character formation. Social learning, emotional development, institutional structure, and broader forms of habituation also contribute to the development of stable conduct. The present claim is more limited: recurrent low-level regulation constitutes an indispensable mechanism through which behavioural continuity is sustained. In this respect, the framework aligns with process-oriented approaches to personality by clarifying how repeated regulation contributes to persistence and gradual trait development.

Micro-discipline may be analysed in terms of four interacting regulatory dimensions.

First, attentional regulation plays a foundational role. Sustained conduct depends on the capacity to repeatedly restore attention following distraction or interruption. Progress often relies less on singular acts of decision than on the repeated re-establishment of focus.

Second, effort continuation sustains activity despite fluctuations in motivation. Many forms of goal-directed behaviour depend on persistence through boredom, fatigue, or resistance rather than on stable desire alone (Hennecke et al., 2019).

Third, impulse modulation protects ongoing activity from fragmentation. Everyday behaviour is frequently disrupted not by major temptations but by low-level forms of friction such as premature task switching, avoidance, or disengagement. Process-based accounts of self-regulation suggest that behavioural continuity often depends on the management of attention, motivation, and situational exposure prior to explicit inhibitory effort (Inzlicht et al., 2014; Milyavskaya et al., 2015). Fourth, behavioural completion stabilises conduct by preserving closure between intention and execution. Completion reduces fragmentation and cognitive residue, whereas repeated non-completion weakens expectations of follow-through.

These dimensions are analytically distinguishable but functionally interconnected. Attentional regulation supports effort continuation, effort supports completion, and impulse modulation protects the stability of all three. Through repetition, these processes gradually reinforce expectations of continuity and increase the stability of organised conduct.

The contribution of the concept lies not in identifying entirely new psychological mechanisms, but in theorising recurrent low-level regulation as a cumulative process through which behavioural patterns become progressively stabilised. Micro-discipline therefore provides a more precise account of how local regulatory processes scale into more enduring forms of behavioural organisation.

### Objections and Clarifications

The explanatory role of micro-discipline becomes clearer when distinguished from adjacent frameworks.

Habit theory explains how repeated behaviour becomes increasingly automatic through contextual cueing and reduced deliberative demand (Wood & Rünger, 2016; Lally et al., 2010). Such accounts describe the transition from effortful action to automatised repetition. Micro-discipline concerns a different aspect of behavioural organisation: the recurrent regulatory processes that sustain conduct prior to full automatization and within domains where complete automatization may never occur.

Self-control research typically focuses on temptation, conflict, and impulse inhibition (Duckworth et al., 2016; Tangney et al., 2004). Research on implementation intentions further demonstrates how pre-specified cue-response structures can support behavioural consistency (Gollwitzer, 1999). Micro-discipline overlaps with this literature but addresses a broader pattern of behavioural maintenance operating even in situations where no salient temptation is present and no explicit plan is actively being executed. Behavioural continuity in such cases still depends on recurrent low-level regulation.

Trait theory, including work on conscientiousness and cybernetic personality structure, describes relatively stable behavioural tendencies and regulatory parameters (McCrae & Costa, 2008; DeYoung, 2015). These approaches are indispensable for characterising behavioural regularities at the dispositional level. Micro-discipline operates at a different explanatory scale. It concerns the repeated enactments through which such regularities are maintained, reinforced, or gradually modified across time.

Virtue ethics addresses the normative organisation of character, including the proper ordering of perception, motivation, and judgment (Aristotle, 1999; Annas, 2011; Snow, 2010). Micro-discipline does not itself constitute virtue. It is better understood as part of the behavioural infrastructure through which commitments, including morally significant ones, become sustainable in practice. Behavioural continuity is also constrained by well-documented limits in self-regulatory capacity and goal maintenance (Baumeister & Heatherton, 1996), which helps explain why organised conduct depends on repeated low-level support rather than on sustained effort alone.

Taken together, these distinctions indicate that micro-discipline is not reducible to habit, self-control, trait structure, or virtue. It identifies a distinct explanatory layer concerned with the recurrent regulatory processes through which behavioural continuity is sustained and more stable patterns of conduct gradually emerge.

## 4. Accumulation, State Distributions, and Behavioural Trajectories

If micro-discipline refers to recurrent low-level regulation that preserves continuity between intention and action, its explanatory significance becomes clearest when behaviour is examined across extended temporal horizons. Individual regulatory acts are typically minor, local, and easily overlooked. Yet when repeated across days, months, and years, they accumulate into recognisable patterns of conduct. 

Character formation therefore exhibits a temporal structure. Behaviour does not unfold simply as a sequence of isolated choices or as the passive expression of fixed dispositions. It develops through repeated cycles of maintenance, interruption, correction, and completion. The central claim of this section is that micro-discipline matters because these recurrent forms of regulation gradually shape the organisation of behaviour across time. In this way, small regulatory successes and failures contribute to more enduring forms of behavioural stability.

Research on everyday self-regulation supports this interpretation, showing that goal pursuit frequently depends on repeated adjustments under conditions of low motivation or aversive task demands rather than on isolated acts of willpower (Hennecke et al., 2019). Contemporary process models similarly describe self-control as unfolding through multiple stages of regulation rather than as a singular event (Duckworth et al., 2016). From this perspective, sustained conduct is not automatic but continuously maintained through ongoing regulatory activity.

If micro-discipline refers to recurrent low-level regulation that preserves continuity between intention and action, its explanatory significance becomes clearest when behaviour is examined across extended temporal horizons. Individual regulatory acts are typically minor and easily overlooked. Yet when repeated across days, months, and years, they can accumulate into recognisable patterns of conduct. Character formation, from this perspective, does not unfold merely through isolated choices or fixed dispositions, but through successive episodes of maintenance, interruption, correction, and completion. The central claim of this section is that recurrent low-level regulation influences the long-range organisation of behaviour by shaping how behavioural patterns persist, fragment, or consolidate across time.

Research on everyday self-regulation supports this interpretation, showing that goal pursuit frequently depends on repeated regulatory adjustments under conditions of low motivation or aversive task demands rather than on isolated acts of willpower (Hennecke et al., 2019). Contemporary process models similarly describe self-control as unfolding through multiple stages of regulation rather than as a singular event (Duckworth et al., 2016). Behavioural coherence, in this view, is not simply given but repeatedly sustained through ongoing regulatory activity.

### 4.1. Small Regulatory Differences and Long-Range Divergence

The cumulative significance of micro-discipline becomes particularly visible when considering individuals who differ only slightly in everyday regulatory consistency. Consider two agents with comparable abilities, aspirations, and opportunities. One tends, with moderate regularity, to restore attention after distraction, complete small obligations, and maintain routines despite minor friction. The other is only somewhat more likely to defer effort, intermittently abandon routines, or allow minor interruptions to redirect activity.

At any single moment, these differences appear trivial. Neither individual is making dramatic decisions, nor do they exhibit sharply contrasting personality profiles. Across repeated episodes, however, small differences in regulatory consistency can gradually produce divergent developmental pathways. One pattern increasingly consolidates reliability, persistence, and follow-through. The other may generate fragmentation in the form of unfinished tasks, unstable routines, and weakened connections between intention and execution.

Traits that later appear as stable characteristics, such as conscientiousness or self-regulatory reliability, may therefore partly reflect the accumulated effects of numerous minor acts of behavioural maintenance. Longitudinal personality research is consistent with this interpretation. Trait change is generally gradual and often associated with ongoing modifications in routines, roles, and behavioural organisation rather than singular transformative events (Roberts et al., 2006, 2008; Hudson & Fraley, 2015).

The claim is not that every minor action is equally consequential or that isolated failures necessarily produce enduring effects. Rather, repeated small differences in regulatory consistency can become developmentally significant when they systematically bias behaviour in particular directions over extended periods of time.

### 4.2. Micro-Discipline and the Distribution of Enacted States

The mechanism underlying these effects can be clarified through process-based approaches to personality. Fleeson’s density-distribution model proposes that traits are best understood as distributions of enacted states across situations and time (Fleeson, 2001). From this perspective, stable traits reflect recurring regularities in how individuals behave, how long particular behavioural modes persist, and how frequently they recur.

Micro-discipline contributes to this process by influencing the recurrence, duration, and interruption of behavioural states. Acts of attentional return increase the likelihood that focused states re-emerge following distraction. Effort continuation extends engagement despite boredom or resistance. Impulse modulation reduces premature interruption by competing demands, while behavioural completion increases the probability that intention-linked activity terminates in execution rather than abandonment.

Recent developments in whole trait theory similarly emphasise that traits emerge through repeated patterns of state enactment distributed across time and context (Jayawickreme et al., 2021). Within this framework, recurrent regulatory activity gradually influences how behavioural states are distributed across episodes by shaping their recurrence and stability.

Over time, these repeated processes can alter behavioural distributions. Certain patterns become more frequent, more stable, and less vulnerable to interruption, while others gradually diminish because they are repeatedly displaced. Micro-discipline therefore does not produce trait change through abrupt transformation. Rather, it incrementally reorganises behavioural probabilities across repeated episodes, eventually contributing to recognisable forms of trait expression.

This process is cumulative and probabilistic rather than deterministic. It also clarifies why the phenomena described here cannot be reduced either to isolated episodes of self-control or to retrospective trait labels. Micro-discipline matters because it shapes the repeated organisation of behaviour through which more stable forms of conduct gradually emerge.

**Figure 1:**
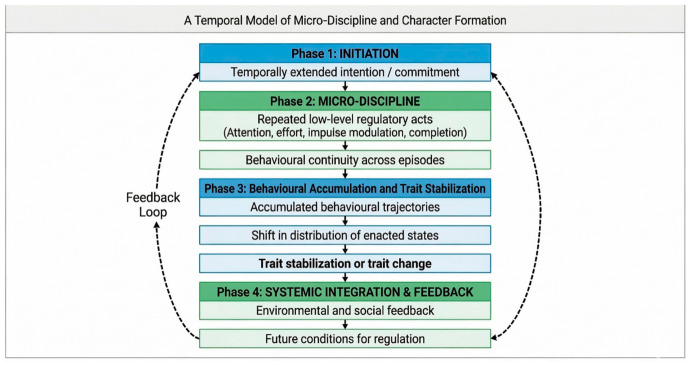
A temporal model of micro-discipline and character formation.

The cumulative relation between recurrent regulation and trait development may therefore be understood as a temporal dynamic in which repeated behavioural maintenance gradually alters the distribution and stability of enacted states (see Figure 1).

The developmental significance of micro-discipline is further amplified by its recursive effects. Repeated follow-through does not simply sustain individual episodes of action; it gradually reshapes the conditions under which subsequent action occurs. Completion reduces backlog and cognitive load, while stable routines reduce the need for repeated deliberation. Consistent follow-through can also strengthen expectations of efficacy and generate trust from others, thereby reinforcing environmental conditions that support organised conduct.

The inverse process is equally important. Repeated fragmentation produces unfinished obligations, cognitive residue, and weakened expectations of follow-through. Irregularity increases the effort required to reinitiate activity, while others may become less willing to rely on the individual, reducing external forms of support that would otherwise facilitate behavioural stability. In this way, behavioural instability can become progressively self-reinforcing.

The figure represents the temporal dynamics of micro-discipline as a recursive system. The initial phase consists of intention-linked behavioural episodes that require regulation under conditions of friction. Repeated low-level regulatory acts stabilise these episodes by preserving continuity between intention and execution. Over time, these stabilised episodes contribute to shifts in the distribution of enacted behavioural states, increasing the recurrence and persistence of certain patterns while reducing others. Environmental and social feedback loops then reshape the conditions under which subsequent regulation occurs, either reinforcing or destabilising behavioural continuity. The model therefore captures both the cumulative and recursive structure of behavioural development.

These recursive dynamics help to explain why micro-discipline has long-term developmental significance despite the apparent triviality of individual acts. Small regulatory adjustments influence not only immediate behaviour but also the future conditions of action. They shape what becomes easier, more probable, and more stable across subsequent episodes. Character formation, in this sense, reflects a gradual process through which behaviour reorganises the conditions of its own recurrence.

### 4.3. A Concrete Illustration: Writing Practice as Behavioural Formation in Miniature

A concrete example helps to illustrate these dynamics. Consider an individual attempting to sustain a writing practice. A simplified explanation might attribute success or failure primarily to motivation, ability, or trait-level conscientiousness. In practice, however, continuity depends on a series of modest regulatory actions: beginning work at the intended time, resisting distraction, persisting through difficulty, returning after interruption, and completing manageable units of work.

Individually, these actions appear insignificant. Missing a session, checking messages, or leaving a paragraph unfinished rarely seems consequential in isolation. Across repeated episodes, however, such decisions gradually shape the organisation of the practice itself. Repeated attentional return, tolerance of discomfort, and task completion increase the recurrence and duration of writing-related behavioural states, whereas postponement, distraction, and premature disengagement reinforce different patterns of conduct.

Over time, these differences may come to be described in dispositional terms. One individual appears reliable or disciplined, while another appears inconsistent. Yet these descriptions follow from the behavioural process rather than explain it. The divergence reflects the cumulative effects of repeated regulatory enactments that either preserve or disrupt alignment between intention and execution.

The example also illustrates why micro-discipline cannot be reduced to habit alone. Certain elements of writing may become routinised, but many aspects remain effortful. The relevant process involves continued regulation under conditions of distraction, uncertainty, fatigue, and fluctuating motivation. It is precisely within such contexts that micro-discipline has explanatory significance.

### 4.4. Path Dependence, Plasticity, and the Conditions of Change

The cumulative effects of micro-discipline imply a path-dependent model of behavioural development. Early patterns of regulation can make later forms of conduct easier or more difficult to sustain. Stable routines may gradually build competence, reliability, and behavioural coherence, whereas repeated fragmentation can undermine continuity and constrain further development.

This does not imply determinism. Behavioural patterns remain revisable, although they are shaped and constrained by prior forms of enactment. Conduct is therefore both durable and plastic: behavioural regularities may become self-reinforcing while remaining open to reorganisation under appropriate conditions.

This perspective bears directly on trait plasticity. Research shows that personality traits can change through intentional behavioural adjustment, although such change is often uneven (Hudson & Fraley, 2015; Hudson & Roberts, 2014). The present framework helps to explain this variability. Trait change depends not only on intention, but on whether intentions are repeatedly translated into regulatory enactment. Without repeated enactment, change efforts fail to accumulate. When stabilised across time, however, repeated regulation can gradually reorganise behavioural distributions and alter trait expression.

Process-oriented models of personality development similarly emphasise that long-term change emerges through repeated cycles linking situational triggers, behavioural states, and feedback processes (Wrzus & Roberts, 2017). Within this framework, micro-discipline may be understood as stabilising the regulatory dimension of these cycles, thereby influencing which behavioural states recur and consolidate across time.

Micro-discipline therefore provides an intermediate explanatory layer between intention and dispositional change, clarifying how momentary regulation gradually scales into more durable forms of behavioural organisation.

## 5. Behavioural Organisation and Normative Evaluation

The preceding analysis has argued that micro-discipline functions as a mechanism in the formation of behavioural stability. Through repeated acts of attentional regulation, effort continuation, impulse modulation, and task completion, individuals gradually construct patterns of conduct that may later appear as stable aspects of character. Behavioural organisation, however, does not by itself constitute moral virtue.

This distinction is necessary because discipline is often treated as inherently admirable. Yet behavioural coherence is ethically neutral. A person may be organised, reliable, and persistent while pursuing harmful or unjust ends. As virtue ethics emphasises, moral evaluation depends not only on behavioural consistency but on the proper ordering of perception, motivation, and judgment (Annas, 2011). Micro-discipline should therefore be understood as a structural capacity that may support, distort, or remain independent of moral virtue.

Maintaining this distinction is central to the present argument. If micro-discipline is to function as an intermediate explanatory concept between action and disposition, it must remain analytically separate from normative evaluation. Its role is to explain how behavioural stability is formed and maintained, not whether it is ethically well directed.

### 5.1. Virtue as Normatively Ordered Agency

Within Aristotelian and neo-Aristotelian ethics, virtue is not merely a pattern of stable behaviour but a normatively ordered disposition involving appropriate perception, rightly formed desire, and sound practical judgment (Aristotle, 1999; Annas, 2011). The virtuous agent does not simply behave consistently, but responds in the right way, for the right reasons, and in proportion to the situation.

This distinction clarifies why behavioural regularity alone is insufficient for virtue. Courage, for example, cannot be reduced to persistence under threat, since one may persist recklessly, stubbornly, or fanatically. What distinguishes courage is the proper ordering of fear, judgment, and purpose. The same applies to temperance, justice, honesty, generosity, and practical wisdom. In each case, the ethical significance of conduct depends on the orientation of the agent’s evaluative and motivational life rather than on behavioural consistency itself.

Contemporary virtue theorists develop this point in complementary ways. Annas emphasises that virtue involves intelligent responsiveness rather than habituated compliance alone. Snow highlights the importance of socially and perceptually informed moral understanding, while Miller argues that moral character cannot be reduced to outward consistency because it depends on a more complex dispositional structure. Taken together, these accounts suggest that virtue is better understood as normatively ordered agency than as behavioural efficiency.

Micro-discipline therefore cannot be equated with virtue without conceptual loss. It is neither equivalent to practical wisdom nor interchangeable with morally ordered habituation. Where virtue concerns the ethical quality of an agent’s orientation toward action, micro-discipline concerns the regulatory processes through which conduct is sustained across time.

### 5.2. Micro-Discipline as Enabling Infrastructure

Although micro-discipline is not itself a virtue, it may function as part of the practical infrastructure through which virtues become behaviourally effective. Moral commitments often fail not because agents reject relevant values, but because the regulatory processes required to sustain them are unstable. Individuals may sincerely endorse honesty, justice, or care while nevertheless failing to act accordingly because attention, effort, or follow-through breaks down.

From this perspective, micro-discipline helps to explain how moral aspiration becomes enacted conduct. The claim is not that virtue reduces to regulation, but that many virtues depend on stable regulatory support as a condition of enactment. As virtue theorists emphasise, moral agency requires not only correct judgment but reliable patterns of action (Annas, 2011). Acts such as sustaining attention to obligations, inhibiting expedient responses, and completing commitments are often indispensable to ethical practice, even when they appear minor in isolation. Research on implementation intentions similarly demonstrates that embedding intentions within structured cues significantly increases behavioural follow-through (Gollwitzer, 1999).

Ethical life is therefore sustained not by evaluative endorsement alone, but through temporally extended patterns of behavioural maintenance. Micro-discipline does not determine moral content, but it helps to explain whether morally significant commitments become stable forms of conduct.

### 5.3. Instrumental Discipline and Moral Orientation

The distinction between micro-discipline and virtue becomes clearer when instrumental discipline is separated from moral orientation. Instrumental discipline refers to the capacity to sustain organised and reliable conduct in the service of an end, whereas moral orientation concerns the normative quality of those ends and the reasoning governing action.

A person may possess considerable behavioural discipline while remaining morally disordered. Precision, reliability, and consistency are not in themselves moral achievements, but forms of behavioural organisation that may serve widely different purposes. As virtue theorists emphasise, moral evaluation depends on the ordering of ends and practical reasoning rather than on behavioural consistency alone (Aristotle, 1999; Annas, 2011). Micro-discipline is therefore ethically neutral: its significance depends on the broader context in which it operates. 

Maintaining this distinction avoids two opposing errors. The first is to treat discipline as inherently admirable. The second is to dismiss it as merely instrumental and philosophically insignificant. A more adequate view is that behavioural organisation matters, although its ethical significance depends on how it is directed.

Micro-discipline does not compete with virtue theory but supplements it. While virtue theory explains the normative structure of character, micro-discipline helps to explain how patterns of action become sufficiently stable for such character to be enacted in practice.

### 5.4. Character Formation and Moral Evaluation

The distinction developed here also clarifies the status of the present theory. Micro-discipline is primarily a descriptive and explanatory concept. It identifies the repeated forms of regulation through which behavioural coherence and dispositional stability gradually emerge. It does not itself provide a criterion of moral worth.

At the same time, the concept has indirect normative significance. Because moral life depends on the reliable enactment of values across time, explanations of behavioural stability remain relevant to ethical theory. As emphasised within virtue ethics, moral agency requires not only correct judgment but stable forms of action (Annas, 2011; Aristotle, 1999). Micro-discipline matters normatively not because it constitutes virtue, but because it helps to explain why virtues become behaviourally durable or fragile.

This point may be stated more directly. Agents often fail to act in accordance with their endorsed values not only because of defective belief, but because behavioural regulation becomes unstable across time. Ethical aspiration without sustained enactment remains vulnerable to interruption and dissipation. Micro-discipline is therefore descriptively primary but normatively consequential, identifying conditions under which moral agency becomes more or less durable.

The framework consequently occupies a middle position. It neither reduces moral evaluation to behavioural efficiency nor treats the mechanics of action as philosophically irrelevant. If ethical theory is concerned with how commitments are enacted in lived time, then the regulatory processes described here become essential for understanding moral formation.

### 5.5. Avoiding Moralised Interpretations of Discipline

A final clarification is necessary because the language of discipline is easily drawn into moralised or ideological interpretations. In contemporary discourse, discipline is often associated with productivity norms, meritocratic self-optimisation, or individualised expectations of self-management. Within such frameworks, behavioural inconsistency is frequently interpreted as evidence of personal failure or insufficient effort. The present account should not be understood in these terms.

Micro-discipline is not a measure of personal worth. Its operation depends partly on environmental conditions shaping the capacity for sustained regulation. Research on self-regulation and cognitive load shows that factors such as stress, scarcity, and competing demands can significantly impair behavioural maintenance (Baumeister & Vohs, 2007; Mullainathan & Shafir, 2013). The ability to sustain organised conduct therefore reflects not only individual effort but also access to stable contexts, supportive structures, and manageable demands.

For this reason, the distinction between behavioural organisation and moral virtue is not merely conceptual. It prevents micro-discipline from collapsing into a framework of individual blame or functioning as a proxy for merit. Properly understood, the concept identifies a dimension of behavioural organisation while leaving open questions concerning moral orientation and structural constraint.

Micro-discipline should therefore be understood neither as a virtue nor as an endorsement of self-optimisation. It is a theoretically specific account of the repeated regulation through which behavioural continuity is sustained, disrupted, or restored. Its ethical significance lies in clarifying that moral life depends not only on what agents value, but also on whether the conditions necessary for sustained enactment are in place.

## 6. Micro-Discipline as an Ecological System

The preceding analysis has described micro-discipline as a recurring process of behavioural regulation. However, accounts that treat regulation as purely internal remain incomplete. The capacity to sustain coherent patterns of action depends not only on individual effort, but also on the social, institutional, and material environments within which behaviour unfolds.

Micro-discipline should therefore be understood as a person–environment regulatory system rather than an exclusively internal capacity. Recurrent forms of regulation are shaped by external scaffolds including routines, deadlines, physical arrangements, and role expectations. What appears as individual discipline is often partially co-produced by environmental structure.

An ecological perspective clarifies that behavioural continuity depends on more than willpower alone and that differences in behavioural stability cannot be attributed solely to character. The conditions supporting sustained regulation are unevenly distributed across contexts.

### 6.1. Environmental Stability and Behavioural Regulation

Sustained behavioural organisation depends partly on stable environments. Routines and self-regulatory practices are easier to maintain when contexts provide predictability, reliable cues, and manageable demands. Work habits, sleep patterns, exercise routines, and focused attention all benefit from temporal regularity and resource stability.

Research on habit formation illustrates this dynamic. Behavioural repetition is facilitated by stable contextual cues, while disruption can fragment even well-established routines (Wood & Rünger, 2016; Lally et al., 2010). This insight extends beyond habit formation being narrowly understood. Even effortful forms of behavioural maintenance depend partly on environmental support, suggesting that the microstructure of agency emerges through interaction between agents and structured contexts.

### 6.2. Distributed Regulation and Cognitive Scaffolding

The ecological dimensions of micro-discipline can be further clarified through theories of distributed cognition and extended agency. Cognitive processes are often partially realised through structures external to the individual, including tools, environments, and institutional arrangements (Clark & Chalmers, 1998). In everyday life, individuals routinely rely on external systems to stabilise memory, attention, and behavioural sequencing.

Calendars, reminders, task lists, deadlines, institutional routines, and shared expectations function as cognitive scaffolds that reduce demands placed on attention and working memory. Rather than continuously reconstructing intentions internally, individuals embed them within external arrangements that help to preserve behavioural continuity across time.

Within this framework, such structures may be understood as supports for micro-discipline. They stabilise behavioural organisation by externalising components of regulation. A calendar entry preserves commitment to a future obligation, a deadline structures completion, and a shared schedule coordinates individual action with collective rhythms.

Behavioural economics provides a complementary perspective. Research on choice architecture demonstrates that relatively small environmental features can systematically shape conduct by influencing the micro-decisions that accumulate across time (Thaler & Sunstein, 2008). Defaults, reminders, prompts, and friction-reducing mechanisms can redirect behavioural patterns without requiring substantial changes in motivation or deliberation. Micro-discipline, from this perspective, is not only enacted but also environmentally structured.

### 6.3. Social Roles, Cognitive Load, and Unequal Support

Social roles do not merely constrain behaviour; they also scaffold it. Life transitions such as entering employment, forming long-term partnerships, or assuming caregiving responsibilities are associated with increases in conscientiousness and emotional stability (Roberts et al., 2008). One interpretation is that such roles impose recurring expectations, stable routines, and forms of external accountability that reinforce repeated regulatory activity.

At the same time, environmental conditions shape micro-discipline by altering cognitive load. Behavioural regulation depends on monitoring, planning, inhibition, and sustained attention, all of which become strained under conditions of uncertainty, chronic stress, financial pressure, or role overload.

Behavioural instability should therefore not be interpreted straightforwardly as evidence of deficient character or insufficient effort. Under conditions of high cognitive or environmental load, fragmentation may reflect situational burden rather than personal failure. Even strong intentions may fail to stabilise conduct when surrounding conditions are persistently disorganising.

The conditions supporting micro-discipline are also unevenly distributed. Access to stable housing, predictable schedules, and manageable cognitive demands varies substantially across contexts, making opportunities for behavioural continuity socially patterned.

### 6.4. Character Within an Ecology of Agency

Integrating these ecological considerations revises the broader account of character developed in this article. Character cannot be understood solely as an internally generated disposition, but must also be understood as the emergent product of repeated regulation occurring within social and material environments that support, channel, or disrupt action.

This ecological dimension is not an optional extension but a condition of explanatory adequacy. Accounts that ignore scaffolding, distributed regulation, and structural conditions risk reducing character to a form of individualised self-management. A more adequate account recognises that behavioural continuity emerges through interaction between regulatory effort and environmental support.

### 6.5. Inequality and the Distribution of Behavioural Conditions

The ecological perspective carries a further implication: the conditions supporting micro-discipline are unevenly distributed. Stable environments, predictable institutional structures, and access to time and cognitive resources are not universally available, meaning that behavioural continuity is shaped partly by structural conditions rather than by individual effort alone.

This has important philosophical consequences. If behavioural stability depends partly on access to supportive scaffolds, then it cannot be attributed solely to virtue, effort, or personal merit. A robust account of micro-discipline must therefore incorporate the unequal distribution of the conditions that make sustained regulation possible.

## 7. The Temporal Structure of Agency

The concept of micro-discipline has implications extending beyond behavioural maintenance alone. It invites reconsideration of the temporal structure of agency itself. Philosophical reflection has often concentrated on two primary poles: moments of deliberative choice and relatively enduring dispositions. The argument developed in this article is that such accounts remain incomplete. Agency also unfolds within an intermediate temporal layer composed of repeated regulatory processes through which behaviour is organised across time.

### 7.1. The Temporal Stratification of Agency

Agency may be understood as temporally stratified. At the shortest temporal scale are episodes of deliberation and decision making. At an intermediate scale are routines, maintenance practices, and recurrent forms of self-regulation. At the broadest scale are relatively durable dispositions and traits that organise conduct across extended periods.

Philosophical accounts of moral agency have often privileged the first scale because decisions are normatively salient. Actions can be evaluated in terms of justification, rationality, or responsiveness to reasons. Personality psychology, by contrast, has frequently emphasised the third scale, where behavioural regularities are summarised in dispositional terms. Yet much of the practical work of character formation occurs at the intermediate level, where behaviour is repeatedly maintained, interrupted, restored, and stabilised across time.

Recognising this intermediate layer clarifies that behavioural stability is neither merely expressed through traits nor generated solely by isolated choices. It emerges through temporally extended processes of regulation distributed across ordinary life.

### 7.2. Agency as Maintenance

A central implication of this framework is that agency should be understood not only as the capacity to initiate action, but also as the capacity to sustain action across time. This reframing shifts attention away from episodic decision making toward the ongoing processes through which intentions remain behaviourally effective.

Models centred primarily on choice risk overestimating moments of deliberation while underestimating the cumulative regulatory work required to preserve continuity between intention and execution. Many failures of agency do not occur at the level of endorsement itself. They emerge through gradual breakdowns in behavioural continuity after decisions have already been made.

This perspective helps to explain a familiar phenomenon: individuals frequently endorse commitments and values that they repeatedly fail to enact. Such failures need not reflect insincerity, ignorance, or defective reasoning. They may instead reflect instability within the regulatory processes necessary for sustaining action across time. Micro-discipline brings this dimension of agency into clearer focus by identifying the mechanisms through which behavioural continuity is preserved, weakened, or restored.

### 7.3. Ethical and Theoretical Implications

Much of moral life depends on temporally extended patterns of conduct. Trustworthiness, reliability, fairness, and care are not established through isolated actions, but through repeated enactment across time. Promises acquire meaning through sustained follow-through. Justice depends partly on continued attentiveness to obligations and the consistent regulation of bias. Responsibility, in practice, is inseparable from the capacity to maintain action under ordinary conditions of friction.

The broader theoretical implications follow directly from this account. By identifying an intermediate temporal layer of agency, the concept of micro-discipline helps to explain how behavioural coherence is constructed, why traits change gradually rather than abruptly, and why virtue requires more than isolated acts of correct judgment. It also clarifies why ecological scaffolding plays a central role in character formation, since behavioural maintenance depends partly on conditions extending beyond the individual.

A theory of character that omits this intermediate layer may successfully describe stable dispositions or evaluate discrete actions, yet still fail to explain how character is practically sustained, disrupted, or transformed in lived time. Micro-discipline contributes precisely at this level by specifying the regulatory processes through which behavioural continuity emerges across temporal scales.

### 7.4. Limitations and Scope Conditions

The present account is conceptual rather than empirical and therefore limited in several respects. First, it does not provide direct empirical validation of micro-disciplinary processes, but instead proposes a theoretical framework intended to guide future investigation. Second, the analysis does not attempt to quantify the relative contribution of micro-disciplinary regulation in comparison with other mechanisms of character formation, including social learning, affective development, and institutional constraint. Third, while the framework identifies an intermediate explanatory layer, it does not model the precise causal pathways through which recurrent regulation contributes to long-term trait change. Finally, the account is not intended as a comprehensive theory of character, but as a clarification of one comparatively underdeveloped process within a broader developmental architecture. Future research may extend this framework through empirical operationalisation, longitudinal analysis, and context-sensitive modelling of behavioural regulation.

## 8. Conclusions

This article has argued that both philosophical and psychological accounts of character tend to emphasise relatively stable outcomes while leaving comparatively underdeveloped the fine-grained regulatory processes through which such stability emerges. In response, the article has introduced the concept of micro-discipline to describe recurrent low-level forms of regulation through which continuity between intention and action is sustained across extended sequences of behaviour.

The contribution does not lie in identifying entirely new psychological mechanisms, but in integrating dispersed processes into a more precise explanatory framework. Micro-discipline specifies how repeated regulatory enactments accumulate across time to contribute to the formation, stabilisation, and modification of behavioural patterns. Without attention to this intermediate explanatory layer, accounts of character remain incomplete in their explanation of how stable forms of conduct are practically maintained.

Behavioural stability, in this account, is neither simply expressed through traits nor generated solely through isolated decisions. It emerges through repeated processes of maintenance, interruption, correction, and completion that gradually organise conduct across time. This perspective helps to explain both why trait expression shifts incrementally rather than abruptly and why sustained patterns of follow-through play a central role in character formation.

The analysis has also emphasised the ecological dimensions of behavioural regulation. Behavioural continuity depends not only on individual effort, but also on routines, social roles, institutional structures, and material environments that support or disrupt sustained regulation. Character formation therefore emerges through interaction between regulatory processes and structured environments rather than through isolated willpower alone.

The theoretical contribution is threefold. First, for virtue theory, the concept of micro-discipline clarifies how moral commitments become behaviourally durable across time. Second, for personality science, it provides a process-oriented account of how repeated enactments stabilise or gradually reshape trait distributions. Third, for research on behavioural change, it highlights the importance of recurrent regulatory maintenance rather than episodic motivation alone.

More broadly, the framework supports a reconceptualisation of agency as temporally extended. Agency is not only a matter of deliberative choice or dispositional structure, but also of sustaining action across time under ordinary conditions of friction. Future research may further examine how micro-disciplinary processes vary across contexts, how environmental scaffolds influence behavioural continuity, and how recurrent regulation contributes to long-term patterns of character development.

## Data Availability

No new data were created or analysed in this study. Data sharing is not applicable.

## References

[B1-behavsci-16-00879] Annas J. (2011). Intelligent virtue.

[B2-behavsci-16-00879] Aristotle, Irwin T. (1999). Nicomachean ethics.

[B3-behavsci-16-00879] Baumeister R. F., Heatherton T. F. (1996). Self-regulation failure: An overview. Psychological Inquiry.

[B4-behavsci-16-00879] Baumeister R. F., Vohs K. D. (2007). Self-regulation, ego depletion, and motivation. Social and Personality Psychology Compass.

[B5-behavsci-16-00879] Clark A., Chalmers D. J. (1998). The extended mind. Analysis.

[B6-behavsci-16-00879] DeYoung C. G. (2015). Cybernetic big five theory. Journal of Research in Personality.

[B7-behavsci-16-00879] Doris J. M. (2002). Lack of character: Personality and moral behavior.

[B8-behavsci-16-00879] Duckworth A. L., Gendler T. S., Gross J. J. (2016). Situational strategies for self-control. Perspectives on Psychological Science.

[B9-behavsci-16-00879] Fleeson W. (2001). Toward a structure- and process-integrated view of personality: Traits as density distributions of states. Journal of Personality and Social Psychology.

[B10-behavsci-16-00879] Gollwitzer P. M. (1999). Implementation intentions: Strong effects of simple plans. American Psychologist.

[B11-behavsci-16-00879] Hennecke M., Czikmantori T., Brandstätter V. (2019). Doing despite disliking: Self-regulatory strategies in everyday aversive activities. European Journal of Personality.

[B12-behavsci-16-00879] Hudson N. W., Fraley R. C. (2015). Volitional personality trait change: Can people choose to change their personality traits?. Journal of Personality and Social Psychology.

[B13-behavsci-16-00879] Hudson N. W., Roberts B. W. (2014). Goals to change personality traits: Concurrent links between personality traits, daily behavior, and goals to change oneself. Journal of Research in Personality.

[B14-behavsci-16-00879] Inzlicht M., Schmeichel B. J., Macrae C. N. (2014). Why self-control seems (but may not be) limited. Trends in Cognitive Sciences.

[B15-behavsci-16-00879] Jayawickreme E., Zachry C. E., Fleeson W. (2021). Whole trait theory: An integrative approach to examining personality structure and process. Personality and Individual Differences.

[B16-behavsci-16-00879] Lally P., van Jaarsveld C. H., Potts H. W., Wardle J. (2010). How are habits formed: Modelling habit formation in the real world. European Journal of Social Psychology.

[B17-behavsci-16-00879] McCrae R. R., Costa P. T., John O. P., Robins R. W., Pervin L. A. (2008). The Five-Factor Theory of personality. Handbook of personality: Theory and research.

[B18-behavsci-16-00879] Milyavskaya M., Inzlicht M., Hope N., Koestner R. (2015). Saying “no” to temptation: Want-to motivation improves self-regulation by reducing temptation rather than by increasing self-control. Journal of Personality and Social Psychology.

[B19-behavsci-16-00879] Mischel W., Shoda Y. (1995). A cognitive-affective system theory of personality: Reconceptualizing situations, dispositions, dynamics, and invariance in personality structure. Psychological Review.

[B20-behavsci-16-00879] Mullainathan S., Shafir E. (2013). Scarcity: Why having too little means so much.

[B21-behavsci-16-00879] Roberts B. W., Walton K. E., Viechtbauer W. (2006). Patterns of mean-level change in personality traits across the life course: A meta-analysis of longitudinal studies. Psychological Bulletin.

[B22-behavsci-16-00879] Roberts B. W., Wood D., Caspi A., John O. P., Robins R. W., Pervin L. A. (2008). The development of personality traits in adulthood. Handbook of personality: Theory and research.

[B23-behavsci-16-00879] Snow N. E. (2010). Virtue as social intelligence: An empirically grounded theory.

[B24-behavsci-16-00879] Tangney J. P., Baumeister R. F., Boone A. L. (2004). High self-control predicts good adjustment, less pathology, better grades, and interpersonal success. Journal of Personality.

[B25-behavsci-16-00879] Thaler R. H., Sunstein C. R. (2008). Nudge: Improving decisions about health, wealth, and happiness.

[B26-behavsci-16-00879] Wood W., Neal D. T. (2007). A new look at habits and the habit–Goal interface. Psychological Review.

[B27-behavsci-16-00879] Wood W., Rünger D. (2016). Psychology of habit. Annual Review of Psychology.

[B28-behavsci-16-00879] Wrzus C., Roberts B. W. (2017). Processes of personality development in adulthood: The TESSERA framework. Personality and Social Psychology Review.

